# Validation of an MLV-based SARS-CoV-2 pseudovirus neutralization assay substantiates L455S-mediated antibody escape

**DOI:** 10.1038/s41598-026-53146-7

**Published:** 2026-05-20

**Authors:** Dominik Moll, David N. Springer, Lukas Weseslindtner, Stephan W. Aberle, Judith H. Aberle, Iris Medits-Weiss, Karin Stiasny

**Affiliations:** Center for Virology, Medical University of Vienna, Vienna, Austria

**Keywords:** SARS-CoV-2, Live-virus neutralization, MLV-based Pseudoviruses, Spike mutation L455S, Immune escape and variant evolution, Diseases, Immunology, Microbiology

## Abstract

The ongoing evolution of SARS-CoV-2 has generated variants with increased transmissibility and immune evasion, primarily driven by mutations in the receptor-binding domain (RBD). Recent Omicron sublineages, including JN.1, have multiple spike substitutions, including L455S, which is associated with antibody evasion and persists in descendant and currently circulating lineages. While live virus neutralization assays (VNTs) remain the gold standard for evaluating immune responses, their biosafety requirements and technical complexity limit large scale application, prompting an increased reliance on pseudovirus-based systems. In this study, we compared murine leukemia virus (MLV)-based pseudovirus neutralization assays (pVNTs) with VNTs across multiple SARS-CoV-2 variants. We also assessed the contribution of the L455S substitution to immune escape, a mutation consistently implicated as a key determinant of JN.1-associated immune evasion. NT titers obtained with the MLV system showed strong correlation with those of live virus assays, supporting its reliability for variant-specific neutralization studies. Introduction of the L455S mutation into two Omicron variants resulted in substantial reductions in neutralization titers, supporting its immune-evasive properties. Together, these findings validate the MLV-based pseudovirus system as a robust NT surrogate and demonstrate its suitability for evaluating emerging mutations linked to variant evolution, such as the L455S mutation, which played a key role in enabling JN.1 to become dominant.

**Supplementary Information:**

The online version contains supplementary material available at 10.1038/s41598-026-53146-7.

## Introduction

The COVID-19 pandemic, caused by SARS-CoV-2, has posed a significant threat to global public health since its emergence in late 2019^1^. The spike protein of SARS-CoV-2 plays a crucial role in viral entry into host cells, with the receptor-binding domain (RBD) interacting with the human angiotensin-converting enzyme 2 (ACE2) receptor^2^. As the pandemic has progressed, SARS-CoV-2 has continuously evolved, giving rise to multiple Variants of Concern (VOCs), including Alpha, Beta, Gamma, Delta, and the highly transmissible Omicron variant and its sublineages. Its spike protein carries numerous mutations, particularly in the RBD region, which significantly enhance viral transmissibility and/or immune escape capabilities^3,4^. BA.2.86, a sublineage of Omicron, carries more than 30 mutations in its spike protein compared to its ancestor, BA.2. JN.1, a descendant of BA.2.86, has acquired additional key mutations, including L455S within the RBD, which has been associated with the rapid global spread of this variant by enhancing immune evasion despite slightly reduced ACE2-binding affinity^5,6^. Subsequent variants have accumulated further mutations in proximity to L455S, thereby improving their ability to evade host immunity^7^. Notably, the continued presence of L455S in multiple descendant lineages and among currently circulating variants points to an important evolutionary advantage. Variants, like KP.2 and KP.3, which belong to the so-called “FLiRT” group, carry this residue and are part of a family of SARS-CoV-2 sublineages derived from JN.1 that share a characteristic set of spike-protein mutations (notably F456L and R346T). These mutations collectively confer enhanced resistance to antibody neutralization and contribute to their ongoing circulation^7,8^. Neutralizing antibodies are a class of antibodies produced by the immune system that can directly block viral entry into host cells. Following SARS-CoV-2 infection or vaccination, assessing neutralizing antibody levels is crucial for understanding immune responses and for guiding booster vaccination strategies. Traditional neutralizing antibody detection methods primarily rely on live virus neutralization tests (NTs), which are considered the gold standard. However, these assays require complex experimental and time-consuming workflows, and high biosafety requirements, which limit their application in large-scale studies. In recent years, pseudovirus systems have emerged as safe and efficient alternatives that have gained widespread application in virology research^9,10^. Pseudoviruses are chimeric viral particles generated by packaging the genome of one virus with the envelope protein of another. They retain the ability to enter host cells but lack replication capacity, thereby significantly reducing biosafety risks. Pseudovirus systems based on Human immunodeficiency virus (HIV) or Vesicular Stomatitis Virus (VSV) backbones have been successfully used in SARS-CoV-2 neutralization studies, demonstrating great potential, particularly for vaccine evaluation, and antiviral drug screening^11–15^. Other systems, like simian immunodeficiency virus (SIV) and Murine leukemia virus (MLV), have been used for neutralization assays with SARS-CoV, while MLV systems have also been applied for Middle East respiratory syndrome coronavirus (MERS-CoV) studies^16–19^. Pseudovirus neutralization assays offer several advantages, including their ability to be performed under Biosafety Level 2 (BSL-2) laboratory conditions and their suitability for high-throughput testing. Moreover, these systems allow flexible and rapid evaluation of the impact of specific mutations on viral neutralization, understanding viral evolution mechanisms, and fast adaptation to emerging variants. Nevertheless, the correlation between pseudovirus NTs and live virus NTs requires systematic validation, especially as new variants continue to emerge. Importantly, this flexibility allows focused analysis of individual spike substitutions that may promote immune escape and/or enhanced transmission dynamics, such as the L455S mutation. While individual aspects of pseudovirus-based neutralization and variant-specific immune escape have been described previously^7,8,11,20–27^, systematic comparisons of MLV-based pseudovirus and live virus neutralization assays across variants, combined with targeted analyses of individual spike mutations, are still limited. The aim of this study was to compare the performance of MLV-based pseudovirus NTs with that of live virus NTs, to evaluate the neutralization profile of different SARS-CoV-2 variants, and to investigate the impact of the single amino acid exchange L455S on neutralizing antibody escape. Our results demonstrate that the MLV-pseudovirus system is highly suitable for analyzing SARS-CoV-2 neutralization and shows excellent correlation with live virus neutralization assays across different genetic backgrounds. The introduction of the L455S mutation significantly reduced neutralization titers across multiple variants, with the largest difference observed between XBB.1.5 and JN.1, indicating a broad impact on antibody recognition that was more pronounced in the XBB.1.5 than in the BA.2.86 background. Together with earlier reports examining whole viral lineages, our findings highlight the functional relevance of an individual spike substitution and demonstrate how adaptable the MLV-pseudovirus system can be for assessing emerging mutations that may influence variant dynamics.

## Results

### Serum samples

This study included 32 human serum samples (Table S1) obtained from individuals with different vaccination and Omicron infection histories (hybrid immunity). The median time since the last immunological event (infection or vaccination) was 33 days (range 7–65 days). 10 pre-pandemic sera were used as negative controls. Detailed information on the vaccination and infection histories as well as neutralization titers obtained in live virus NTs (VNTs) and pseudovirus NTs (pVNTs) are provided in the Supplementary Table S2.

### Pseudotyped and live virus neutralization assays show strong correlation

**Fig. 1:**
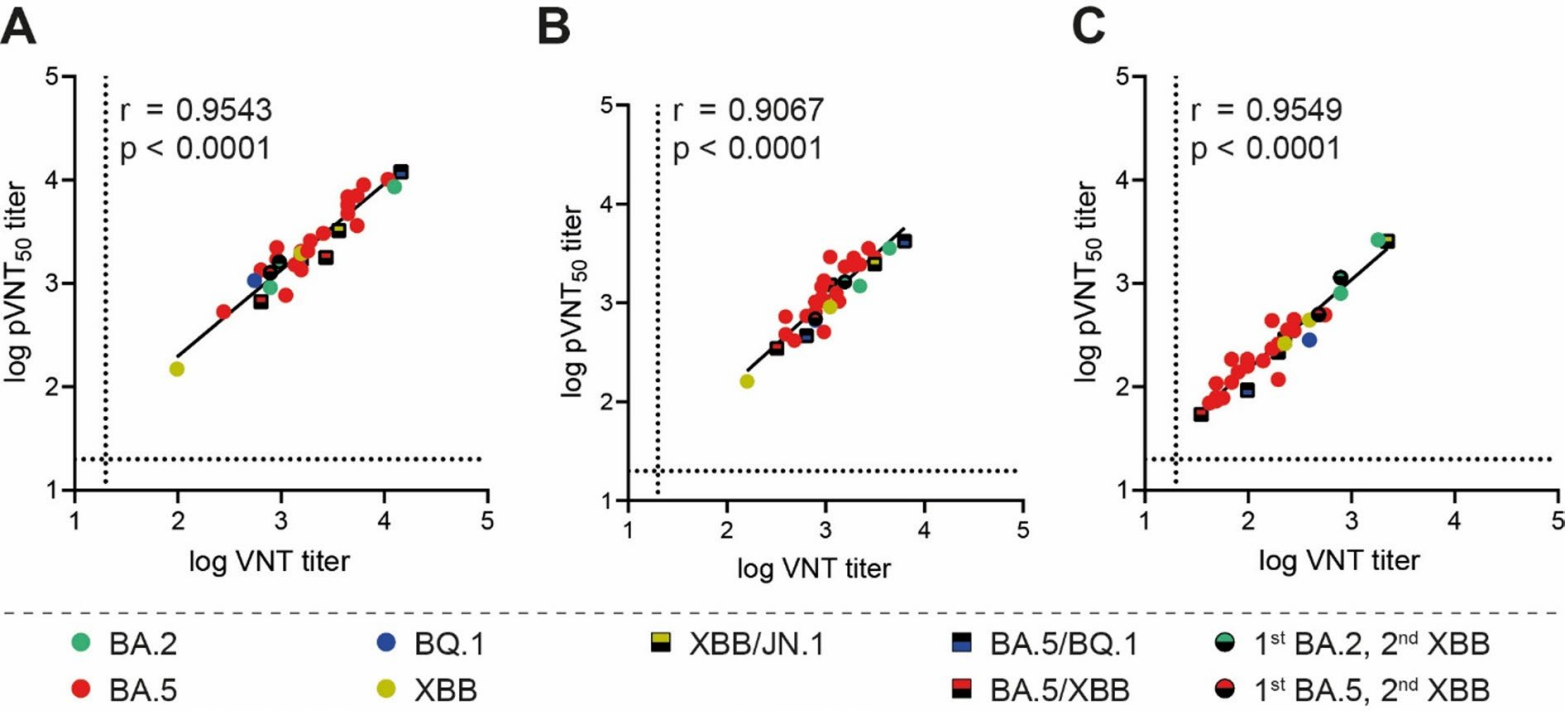
Pearson´s correlation of geometric mean neutralization titers measured by pVNT and VNT against SARS-CoV-2 variants. Correlation between log10-transformed serum neutralization titers against SARS-CoV-2 D614G **(A)**, BA.5 **(B)**, and XBB.1.5 **(C)** as determined by pseudovirus neutralization tests (pVNTs) and live virus neutralization tests (VNTs). A total of 32 serum samples were analyzed in both assays. Breakthrough infection variants are indicated by color and symbol. Single BTIs are shown as fully colored circles; co-circulating–variant infections as half-black, half-colored squares; and first and second infections as half-black, half-colored circles (see Table S1 and S2). Dashed lines indicate the cut-off threshold. Data are from at least three independent experiments. Pearson correlation coefficients (r) and p values are indicated.

We established MLV-based pseudovirus NTs (pVNTs) incorporating the D614G, BA.5, and XBB.1.5 variants with a luciferase reporter read-out. To assess the performance of our pVNTs with the different variants, we compared it with the corresponding live virus neutralization tests (VNTs) based on a cytopathic effect (CPE) read-out for infectivity. Testing of 32 human serum samples revealed that the two methods were highly consistent in detecting neutralizing antibodies against the three variants (Fig. 1). The correlation coefficient r between pVNT and VNT was ≥ 0.90 for all variants tested, indicating excellent agreement between the two methods. In addition, 10 pre-pandemic serum samples were tested negative in the pVNT, confirming assay specificity (Figure S1). These results strongly demonstrate the suitability of our pseudovirus NT as an alternative to live virus NTs.

**Fig. 2:**
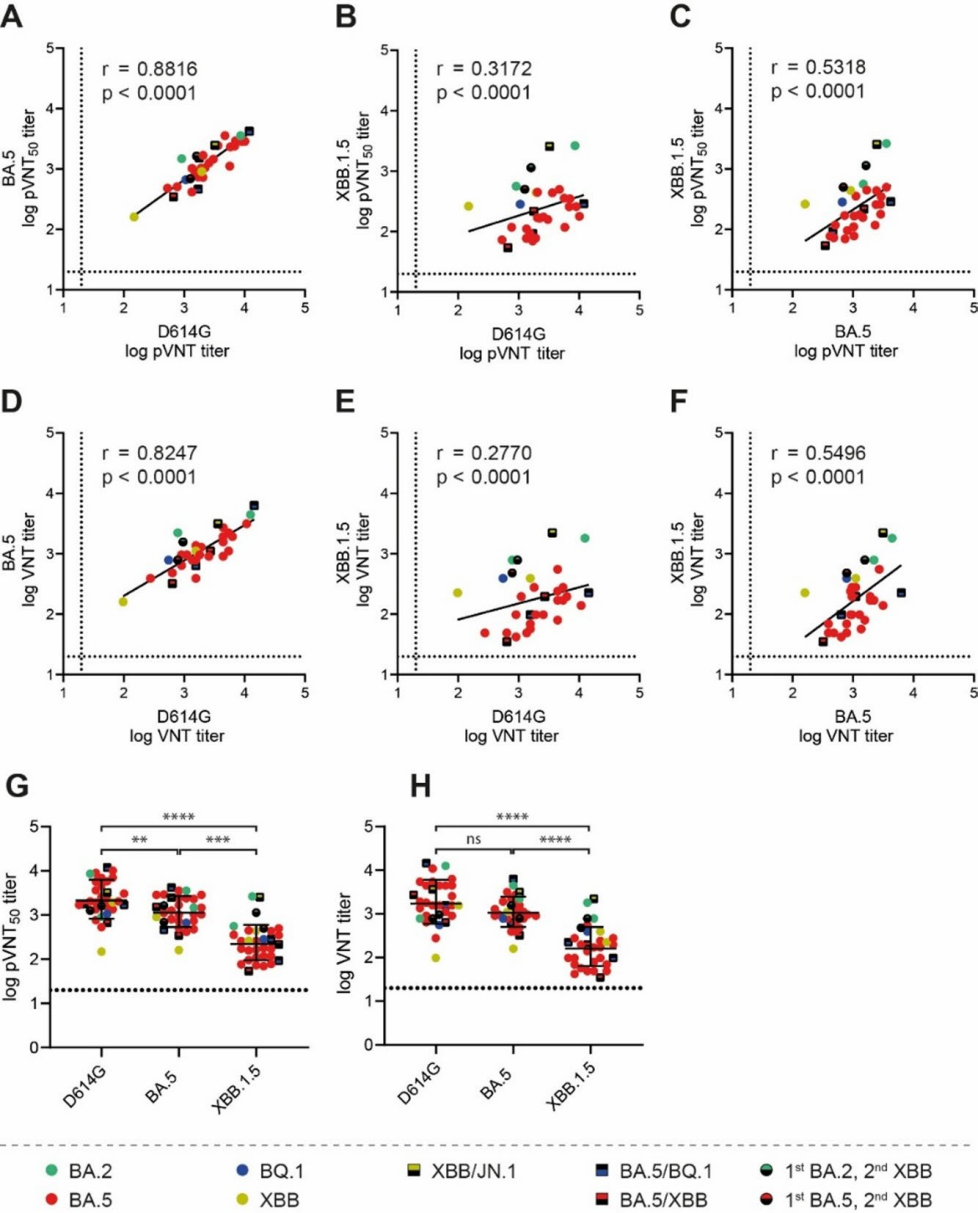
Pearson´s correlation and geometric mean neutralization titers measured by pVNT and VNT against SARS-CoV-2 variants. **(A-F)** Correlation of log10-transformed serum neutralization titers between SARS-CoV-2 D614G and BA.5 **(A**,** D)**, D614G and XBB.1.5 **(B**,** E)**, and BA.5 and XBB.1.5 **(C**,** F)** as determined by pseudovirus neutralization tests (pVNTs; A-C) and live virus neutralization tests (VNTs; D-F). A total of 32 serum samples were analyzed in both assays. Breakthrough infection variants are indicated by color and symbol. Single BTIs are shown as fully colored circles; co-circulating–variant infections as half-black, half-colored squares; and first and second infections as half-black, half-colored circles (see Table S1 and S2). Dashed lines indicate the cut-off threshold. Data are from at least three independent experiments. Pearson correlation coefficients (r) and p values are indicated.(G, H) Serum neutralization titers of SARS-CoV-2 variants measured by pVNTs **(G)** and VNTs **(H)**. Data are presented as geometric mean titers (GMT) ± SD. Statistical significance was assessed using a Friedman test with Dunn’s multiple comparisons test for paired samples. Serum samples with a log10 titer of 1 were considered non-neutralizing. Dashed horizontal lines indicate the cut-off threshold. Asterisks indicate significance thresholds (** *p* < 0.01, *** *p* < 0.001, **** *p* < 0.0001); ns, not significant.

When comparing the neutralization titers of SARS-CoV-2 variants (Fig. 2), we observed a strong positive correlation between D614G and BA.5 (Fig. 2A, D) as well as a moderate correlation between BA.5 and XBB.1.5 (Fig. 2C, F). In contrast, the correlation between XBB.1.5 and D614G (Fig. 2B, F) was weak, although still statistically significant. Comparable patterns were observed in the VNTs and pVNTs. Overall, neutralization titers were higher against D614G than the Omicron variants in both pVNTs and VNTs (Fig. 2G and H).

### The L455S mutation significantly reduces virus neutralization

To understand the contribution of the L455S mutation to neutralizing antibody escape, we used our pVNT to directly assess if the L455S mutation can alter neutralization activities. Using the XBB.1.5 variant, we generated an XBB.1.5 + L455S pseudovirus and compared its neutralization profile with the parental strain. Neutralization assays were performed using 31 of the 32 serum samples due to insufficient volume of one sample. Because XBB lineages have been largely replaced by JN.1 in circulation, we additionally established BA.2.86 and JN.1. Notably, JN.1 is derived from BA.2.86 and differs by a single spike mutation (L455S)^5,6^, enabling a direct assessment of the effect of this mutation across variant backgrounds.

**Fig. 3:**
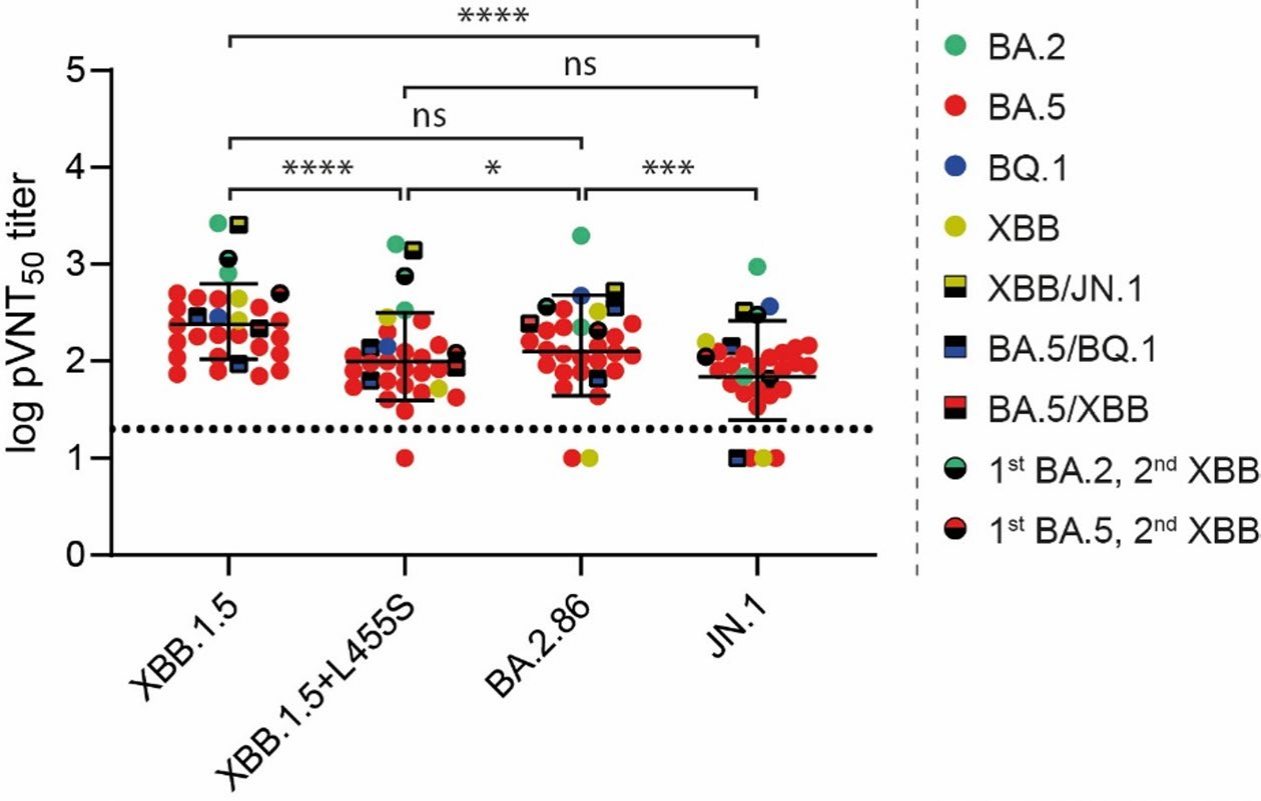
Serum neutralization of SARS-CoV-2 variants measured by pseudovirus neutralization assays. Serum neutralization titers against SARS-CoV-2 XBB.1.5, XBB.1.5 + L455S, BA.2.86, and JN.1 (BA.2.86 with the L455S mutation) as determined by pseudovirus neutralization tests (pVNTs; *n* = 31 serum samples). Data are presented as geometric mean titers (GMT) ± SD. Statistical significance was assessed using a Friedman test with Dunn’s multiple comparisons test for paired samples. Serum samples with a log10 pVNT₅₀ titer of 1 were considered non-neutralizing. Dashed horizontal line indicates the cut-off threshold. Asterisks indicate significance thresholds (* *p* < 0.05, *** *p* < 0.001, ****p < 0.0001); ns, not significant.

Introduction of the L455S mutation into the XBB.1.5 variant resulted in a significant 2.3-fold reduction in geometric mean neutralization titers (GMT), demonstrating substantial antibody escape. A significant reduction was also observed between XBB.1.5 and JN.1. (GMT, 3.2-fold). However, no significant difference was observed compared to BA.2.86, highlighting the broad effect of the L455S substitution on neutralizing antibody recognition (Fig. 3). Notably, the effect of L455S was slightly more pronounced in the XBB.1.5 background than in the BA.2.86. background (2.3- and 1.8-fold GMT reduction, respectively).

Despite significant differences in neutralization potency caused by the mutation, we found that the overall neutralization patterns highly correlated across all four variants (*r* > 0.8; Fig. S2).

## Discussion

In this study, we demonstrate high consistency between murine leukemia virus (MLV)-based pseudovirus neutralization tests (pVNTs) and live virus neutralization tests (VNTs) in detecting SARS-CoV-2 neutralizing antibodies across a broad range of serum samples collected during different variant waves. Neutralization titers obtained with both assay systems showed excellent correlation, as reported for other and similar systems^11^. Notably, comparable neutralization titers were observed in both assay systems despite using different viral systems and target cell lines (Vero E6 in VNTs versus HEK293T-ACE2-TMPRSS2 in pVNTs).

Our findings align with multiple previous studies demonstrating strong agreement between pseudovirus and live virus neutralization assays. For example, a high-throughput, automated pseudovirus neutralization assay based on Vesicular Stomatitis Virus (VSV) showed good correlation with live virus neutralization tests^28^. In addition, pseudovirus systems based on lentiviral/HIV backbones have also been reported to be well suited for such analyses and to exhibit similarly high correlation^13–15^. A comprehensive meta-analysis further supported these observations by showing high consistency between pseudovirus and live virus neutralization assays across studies, assay formats, and SARS-CoV-2 variants^11^, although MLV-based systems were underrepresented. Later studies have also continued to validate the reliability of both VSV-based^9^ and lentiviral/HIV-based pVNTs^21,29^.

In contrast to these well-studied approaches, the pseudovirus system used in the present study is based on the MLV backbone. Although a previous report demonstrated strong agreement between an MLV-based pVNT and live virus assays^20^, this backbone has been less systematically evaluated, particularly in the context of emerging SARS-CoV-2 variants, and the available evidence remains limited compared to VSV- and lentiviral-based systems.

Our study directly addressed this gap by systematically comparing a standardized MLV-based pVNT with VNTs using serum samples across multiple variants, demonstrating robust agreement across a wide dynamic range of neutralization titers and providing further evidence for the suitability of MLV systems for high-throughput neutralization assays. In addition, we combine this approach with a targeted analysis of a single spike mutation (L455S) across different variant backgrounds, enabling a more refined assessment of mutation-driven immune escape.

In this context, the choice of pseudotype system can influence assay performance and interpretation. An MLV-based pseudotype system provides a robust and technically straightforward platform with a favorable safety profile and reliable virus production, and can generate high viral yields compared to lentiviral systems^30^. VSV-based pseudotypes may enable faster reporter expression but can be associated with increased cytotoxicity and residual helper virus background^10^, whereas lentiviral systems are often more complex to handle. Although all systems are limited to modeling viral entry rather than the full replication cycle^11,31^, the strong correlation observed between the MLV-based pseudovirus assay and live virus neutralization across variants in our study supports the suitability of this assay for quantitative neutralization analyses and for evaluating mutation-driven immune escape.

Taken together, our results extend established pseudovirus neutralization systems to include MLV-based systems that may offer practical advantages for laboratory use, including straightforward production workflows, flexible spike incorporation, and suitability for scalable variant testing.

However, it is also important to note that pseudovirus systems have certain limitations. Specifically, these systems can only simulate viral entry processes and cannot fully replicate the complete life cycle of a live virus within cells. Additionally, different pseudovirus systems may vary in packaging efficiency and infection characteristics^17,30^. Nevertheless, pseudoviruses remain valuable research tools, but designing experiments, interpreting, and comparing results must be done carefully.

As noted above, these systems are well suited to assess the impact of individual spike mutations on immune escape. Using our approach, we analyzed the effect of the L455S mutation, a characteristic mutation of the JN.1 variant, compared with its ancestral BA.2.86 lineage, which has been reported to be responsible for significant neutralization escape and rapid global spread^24^. Notably, L455S has persisted across multiple descendant lineages and remains prevalent among currently circulating variants, suggesting a sustained selective advantage.

Consistent with our findings, Zhang et al. used a VSV-based pseudovirus assay in TMPRSS2-expressing cells with sera from vaccinated individuals and showed that JN.1, characterized by the L455S mutation, was less well neutralized than BA.2.86^6^. Li et al. similarly used a lentivirus-based pseudovirus system in ACE2-expressing cells with vaccinated and convalescent sera and reported strong antibody evasion by JN.1 and subsequent lineages^8^. Liu et al. further demonstrated broad neutralization escape of emerging Omicron variants using lentivirus-based pseudovirus and live virus assays with BA.4/5-convalencent sera and monoclonal antibodies^32^. We found that the introduction of L455S, either in the JN.1 background or into an XBB.1.5 backbone, markedly reduced neutralization titers in sera collected during the XBB.1.5 wave before JN.1 became dominant, supporting a key role for this substitution in antibody escape and, likely, in the spread of JN.1. The stronger effect of L455S in the XBB.1.5 background compared to BA.2.86 may reflect differences in baseline neutralization. While BA.2.86 already exhibits substantial immune escape from BA.5-elicited antibodies, variants such as XBB.1.5 may remain partially susceptible, allowing individual substitutions to have a more pronounced effect on antibody recognition. This is consistent with previous reports describing enhanced immune evasion of JN.1 compared to earlier Omicron subvariants^33–37^.

Data from JN.1 and related L455S-containing subvariants suggest that adding this mutation is associated with enhanced immune evasion, likely by reducing susceptibility to neutralizing antibodies, even if it affects ACE2 binding affinity and other virological properties^5–8,22,24^. Although the assay systems and target cells differed, all studies support a key role for L455S in JN.1-associated immune escape. Our data further underscore the functional relevance of this single spike substitution and demonstrate how rapidly adaptable pseudovirus systems can be applied to evaluate emerging mutations that may contribute to variant replacement.

In conclusion, the strong correlation between MLV-based pseudovirus neutralization and live virus neutralization assays, combined with the influence of key spike mutations on antigenicity, underlines the continued relevance of pseudovirus systems in SARS-CoV-2 research and pandemic response. This combined approach provides a framework to evaluate emerging SARS-CoV-2 variants and their immune escape.

### Limitations of the study

The number of serum samples analyzed was limited, which may restrict the generalizability of the neutralization findings. As with many cohort-based studies, the infection histories of the participants and the vaccine formulations represented may not fully capture the current epidemiological landscape. In addition, undiagnosed or asymptomatic infections prior to Omicron cannot be excluded and may have influenced the observed neutralization responses. Furthermore, older variants of concern were included primarily for proof-of-principle comparisons and do not reflect strains that are currently dominant.

## Materials & methods

### Serum samples and ethical approval

This study included 32 human serum samples obtained from individuals with different vaccination and Omicron infection histories (hybrid sera). 10 pre-pandemic sera were used as negative controls. Serum samples were residual diagnostic material sent to the Center for Virology, Medical University of Vienna, and were used in an anonymized manner. The use of anonymized residual diagnostic material was approved by the Ethics Committee of the Medical University of Vienna, Austria (EK1291/2021). The requirement for written informed consent was waived in accordance with national legislation and institutional requirements. All methods were carried out in accordance with relevant guidelines and regulations and the Declaration of Helsinki. Detailed cohort information is provided in Table S2. A subset of the serum panel included in this study has been reported previously^37,38^. Breakthrough infections (BTIs) were confirmed by PCR, and the infecting variant was assigned when possible. In five cases, samples were collected during periods of co-circulating variants and could not be unambiguously assigned, as indicated in Table S2. These assignments were based on epidemiological data, and sequencing data were not available for all samples.

### Cell lines

HEK293T cells (ATCC CRL-3216) were maintained in Dulbecco’s Modified Eagle Medium (DMEM, Gibco) supplemented with 10 mM HEPES (Serva), 10% heat-inactivated fetal bovine serum (FBS, Capricorn), and 1% penicillin–streptomycin–glutamine (PSG, Cytiva) at 37 °C in a humidified incubator with 5% CO₂. Modified HEK293T cells stably expressing angiotensin-converting enzyme 2 and transmembrane protease serine 2 (HEK293T-ACE2-TMPRSS2; BioCat GmbH, #SL222) were cultured under the same conditions in DMEM containing 10 mM HEPES, 10% heat-inactivated FBS, 1% PSG, 1 µg/mL puromycin, and 100 µg/mL hygromycin B. Vero E6 cells (Sigma-Aldrich, # ECACC 85020206) were cultured in DMEM supplemented with 10% heat-inactivated FBS and 1% PSG at 37 °C in a humidified atmosphere containing 5% CO₂.

### Plasmid design and mutagenesis

To generate SARS-CoV-2 pseudotypes, three plasmids containing codon-optimized sequences encoding the SARS-CoV-2 spike protein (S), a luciferase reporter gene (pTG-Luc), and the Murine Leukemia Virus (MLV) backbone proteins (pCMV-gagpol) were co-transfected into HEK293T cells (ATCC CRL-3216). The pTG-Luc and pCMV-gagpol plasmids were kindly provided by Dr. Jean Kaoru Millet^17^. The pCMV-gagpol plasmid encodes the gagpol polyprotein, which is processed into the capsid, nucleocapsid, and matrix proteins (gag) and the reverse transcriptase (pol), under the control of a cytomegalovirus (CMV) promoter. Spike open reading frames were cloned into a pcDNA3.1(+) vector (Invitrogen). Plasmids encoding the ancestral D614G spike and the variants XBB.1.5 and JN.1 were obtained from GeneArt Thermo Fisher Scientific. Plasmids encoding BA.5, BA.2.86, and XBB.1.5 + L455S spike proteins were generated by site-directed mutagenesis PCR using templates containing the BA.2, JN.1, or XBB.1.5 spike sequences, following the manufacturer’s instructions (Promega). An ampicillin resistance gene was included to allow for bacterial selection (MAX Efficiency DH5α Competent Cells, Invitrogen), and successful mutagenesis was confirmed by Sanger sequencing.

### Generation of pseudotyped virus particles

One day prior to transfection, 1.2 × 10⁷ HEK293T cells were seeded in 16 mL of DMEM (supplemented with 10 mM HEPES, 10% FBS, and 1% PSG) in a T-75 cm² flask and incubated overnight at 37 °C with 5% CO₂. On the day of transfection, the medium was replaced with 8 mL Opti-Minimum Essential Medium (Opti-MEM, Gibco). Two mixtures were prepared and incubated separately at room temperature (RT) for 5 min. The first mixture contained plasmids encoding the luciferase reporter gene and the full-length SARS-CoV-2 spike protein (each 2,400 ng) along with 3,200 ng of the plasmid encoding the Murine Leukemia Virus (MLV) gagpol backbone. The second mixture contained Lipofectamine 2000 (Invitrogen), prepared according to the manufacturer’s instructions. After combining the two mixtures and incubating for 20 min at RT, the transfection complex was added dropwise to the cells. Following 4–6 h of incubation at 37 °C with 5% CO₂, the medium was replaced with DMEM (supplemented with 10 mM HEPES and 10% FBS), and cells were incubated for an additional 48 h. Pseudotyped virus particles were harvested by centrifuging the supernatant at 290 × g for 7 min and sterile-filtering through a 0.45 μm membrane. Aliquots were stored at − 80 °C.

### Pseudovirus neutralization tests (pVNT)

96-well plates were coated with 50 µL of a 1:1 poly-D-lysine/PBS (pH 7.4, Gibco) solution for 1 h at room temperature (RT) and washed three times with ddH₂O. HEK293T-ACE2-TMPRSS2 (BioCat, SL222) cells were then seeded at 1 × 10⁶ cells/mL in 100 µL per well and incubated overnight at 37 °C with 5% CO₂. The next day, cells were washed three times with pre-warmed PBS, and pseudovirus preparations were mixed with 1:2 serially diluted sera (starting at a final dilution of 1∶20) in DMEM (supplemented with 10 mM HEPES, 2% FBS, and 1% PSG) and incubated for 1 h at 37 °C. Fifty microliters of the virus–serum mixtures were added per well and incubated for 1–2 h, after which 50 µL of DMEM (10 mM HEPES, 10% FBS, 1% PSG) was added. Plates were incubated for 72 h before washing once with PBS and performing the luciferase assay following the manufacturer’s instructions (Promega). Luminescence was measured using a Synergy HTX microplate reader (BioTek), and infection levels were quantified as relative light units (RLU).

### Infectious virus neutralization tests (VNT)

Live virus neutralization tests were conducted in Biosafety Level 3 laboratories using Vero E6 cells (Sigma-Aldrich, #ECACC 85020206), as described previously^39^. Cells were cultured in DMEM supplemented with 10% heat-inactivated FBS and 1% PSG. Serum samples were heat-inactivated for 30 min at 56 °C and were serially diluted (starting dilution 1:10). Equal volumes of diluted sera were mixed with 50–100 TCID₅₀ of virus strains D614G (GISAID EPI_ISL_438123), BA.5 (GISAID EPI_ISL_15982848), and XBB.1.5. (GISAID EPI_ISL_17062381) and incubated for 1 h at 37 °C (starting final dilution of sera 1∶20) before addition of Vero E6 cells. All sera were tested in duplicates. Virus-only and cell-only controls were included on each plate. After 5–7 days incubation at 37 °C, cytopathic effect (CPE) was observed by light microscopy, and neutralization titers were defined as the serum dilution that protected cells from viral infection. NT titers ≥ 20 were considered positive.

### Statistical analyses

Statistical analyses were performed using GraphPad Prism 10.2.3 (GraphPad Software Inc.). Pearson correlation analyses on log-transformed data were used to evaluate the relationship between neutralization titers obtained with live SARS-CoV-2–based assays and those obtained with the pseudovirus neutralization assay. Neutralization titers between different SARS-CoV-2 strains or mutants were compared using Friedman test with Dunn’s multiple comparisons test for paired samples.

During the preparation of this manuscript, generative AI tools (ChatGPT 5.2/DeepL/Grammarly Pro) were used for grammatical refinement and suggest phrasing for better readability. All AI-generated suggestions were critically reviewed and verified by the authors. No generative AI was used to create the original text or the scientific arguments.

## Electronic Supplementary Material

Below is the link to the electronic supplementary material.

Supplementary Material 1

## Data Availability

The datasets generated and/or analyzed during the current study will be available from the corresponding authors upon request, Iris Medits-Weiss ([iris.medits-weiss@meduniwien.ac.at](mailto: iris.medits-weiss@meduniwien.ac.at)) and Karin Stiasny ([karin.stiasny@meduniwien.ac.at](mailto: karin.stiasny@meduniwien.ac.at) **)**.
