# Supplementary material for: Validation of an MLV-based SARS-CoV-2 pseudovirus neutralization assay substantiates L455S-mediated antibody escape
Source: Sci Rep. 2026 May 20;16:22881. doi: 10.1038/s41598-026-53146-7 (PMC13389565; doi:10.1038/s41598-026-53146-7)
Supplement: Supplementary file 1 — Supplementary Material 1 [file 41598_2026_53146_MOESM1_ESM.docx]

**SUPPLEMENTARY INFORMATION**

**Table S1:** Number of serum samples per breakthrough infection (BTI) variant.

| BTI variant | Nbr. of sera |  |
| --- | --- | --- |
| BA.2^1^ | 2 |  |
| BA.5^1^ | 20 |  |
| BQ.1.1^1^ | 1 |  |
| XBB^1^ | 2 |  |
| XBB/JN.1^2^ | 1 |  |
| BA.5/BQ.1^2^ | 2 |  |
| BA.5/XBB^2^ | 2 |  |
| 1^st^ BA.2, 2^nd^ XBB^3^ | 1 |  |
| 1^st^ BA.5, 2^nd^ XBB^3^ | 1 |  |
| ^1^ Single-variant infections (BA.2, BA.5, BQ.1.1, XBB) | | |
| ^2^ Infections during waves with co-circulating variants where the infecting lineage could not be clearly assigned (XBB/JN.1, BA.5/BQ.1.1, BA.5/XBB) | | |
| ^3^ Sequential infections indicating first and second infecting variants (1^st^ BA.2, 2^nd^ XBB; 1^st^ BA.5, 2^nd^ XBB) | | |


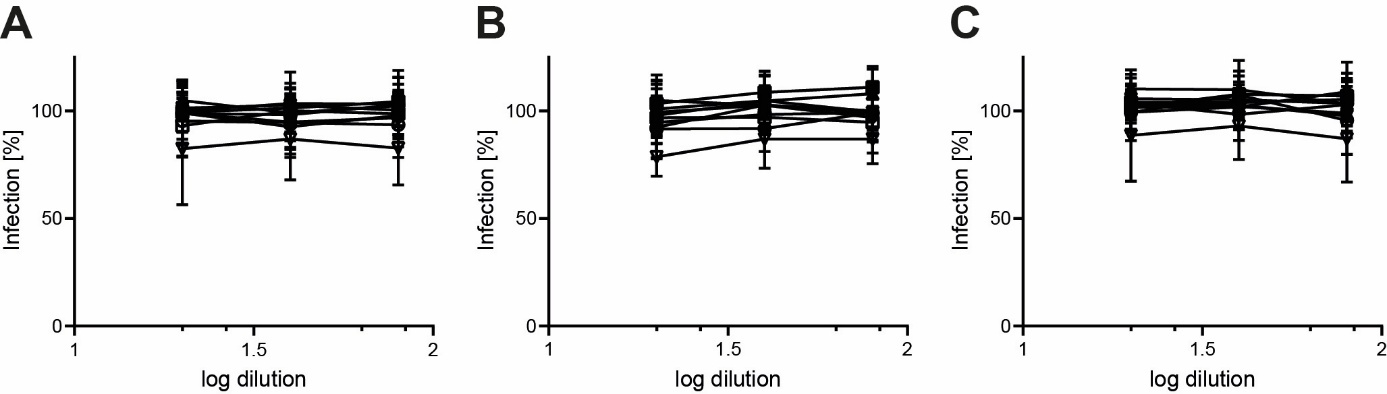


**Figure S1: Pseudovirus neutralization test (pVNT) titration of negative serum samples against different SARS-CoV-2 spike variants.**

Percentage of infection for negative control sera tested against SARS-CoV-2 D614G (A), BA.5 (B), and XBB.1.5 (C) tested in the pVNT. 10 pre-pandemic serum samples were analyzed. Data are from at least three independent experiments. Infection values above 125% were set to 100% for visualization.

**Table S2: Serum samples**

| Serum | BTI variant | | Vaccination history | Age  (years) | Sex (f/m) | dpV^1^ | dpI^2^ | GMT (pVNT) | | | | | | GMT (VNT) | | |
| --- | --- | --- | --- | --- | --- | --- | --- | --- | --- | --- | --- | --- | --- | --- | --- | --- |
|  |  |  |  |  |  |  |  | D614G | BA.5 | XBB.1.5 | BA.2.86 | JN.1 | XBB.1.5+L455S | D614G | BA.5 | XBB.1.5 |
| 1 | BA.2^1^ | | 3x WT, 1x XBB | 30 | f | 32 | 679 | 910 | 1476 | 805 | 223 | 69 | 334 | 232 | 1476 | 805 |
| 2 | BA.2^1^ | | 4x WT, 1x XBB | 25 | m | 53 | n.d. | 8586 | 3566 | 2642 | 1963 | 943 | 1615 | 1698 | 3566 | 2642 |
| 3 | BA.5^1^ | | 4x WT, 1x XBB | 58 | m | 38 | 452 | 2051 | 1686 | 448 | 223 | 122 | 146 | 230 | 1686 | 448 |
| 4 | BA.5^1^ | | 2x WT | 36 | f | 289 | 29 | 532 | 482 | 73 | 10 | 10 | 10 | 19 | 482 | 73 |
| 5 | BA.5^1^ | | 3x WT | 45 | m | 287 | 28 | 1517 | 808 | 108 | 53 | 10 | 31 | 38 | 808 | 108 |
| 6 | BA.5^1^ | | 3x WT | 36 | f | 283 | 41 | 1347 | 417 | 79 | 77 | 44 | 40 | 65 | 417 | 79 |
| 7 | BA.5^1^ | | 3x WT | 39 | f | 248 | 48 | 1709 | 740 | 70 | 91 | 137 | 113 | 95 | 740 | 70 |
| 8 | BA.5^1^ | | 3x WT | 28 | f | 290 | 7 | 769 | 508 | 118 | 159 | 89 | 47 | 119 | 508 | 118 |
| 9 | BA.5^1^ | | 3x WT | 51 | f | 299 | 14 | 1911 | 1036 | 78 | 122 | 80 | 56 | 91 | 1036 | 78 |
| 10 | BA.5^1^ | | 3x WT | 58 | f | 284 | 22 | 6883 | 2406 | 233 | 206 | 82 | 62 | 158 | 2406 | 233 |
| 11 | BA.5^1^ | | 3x WT | 58 | m | 268 | 25 | 8987 | 2851 | 259 | 343 | 108 | 75 | 212 | 2851 | 259 |
| 12 | BA.5^1^ | | 3x WT | 59 | m | 280 | 25 | 5678 | 1112 | 357 | 142 | 89 | 42 | 166 | 1112 | 357 |
| 13 | BA.5^1^ | | 3x WT | 53 | m | 295 | 29 | 7053 | 2911 | 349 | 114 | 46 | 95 | 122 | 2911 | 349 |
| 14 | BA.5^1^ | | 3x WT | 31 | f | 287 | 29 | 3058 | 1457 | 158 | 109 | 95 | 82 | 118 | 1457 | 158 |
| 15 | BA.5^1^ | | 3x WT | 53 | f | 276 | 30 | 10165 | 2865 | 179 | 130 | 90 | 79 | 128 | 2865 | 179 |
| 16 | BA.5^1^ | | 3x WT | 50 | m | 249 | 33 | 2582 | 1263 | 174 | 118 | 116 | 108 | 134 | 1263 | 174 |
| 17 | BA.5^1^ | | 3x WT | 31 | f | 331 | 53 | 5801 | 2320 | 139 | 100 | 58 | 82 | 93 | 2320 | 139 |
| 18 | BA.5^1^ | | 3x WT | 61 | m | 286 | 46 | 2223 | 1042 | 185 | 79 | 34 | 99 | 80 | 1042 | 185 |
| 19 | BA.5^1^ | | 3x WT | 34 | m | 270 | 38 | 2042 | 727 | 185 | 43 | 51 | 124 | 74 | 727 | 185 |
| 20 | BA.5^1^ | | 3x WT | 51 | f | 288 | 57 | 4732 | 3565 | 498 | 242 | 124 | 199 | 246 | 3565 | 498 |
| 21 | BA.5^1^ | | 3x WT | 33 | m | 279 | 57 | 3631 | 2446 | 437 | 177 | 144 | 262 | 223 | 2446 | 437 |
| 22 | BA.5^1^ | | 3x WT | 44 | f | 282 | 65 | 1357 | ≥24 | 111 | 76 | 51 | 54 | 76 | 1024 | 111 |
| 23 | BQ.1.1^1^ | | 4x WT | 56 | f | 42 | 373 | 1065 | 666 | 284 | 473 | 367 | 141 | 367 | 666 | 284 |
| 24 | XBB^1^ | | 4x WT, 1x biv.BA.5 | 40 | m | 334 | 34 | 1957 | 908 | 442 | 324 | 156 | 283 | 282 | 908 | 442 |
| 25 | XBB^1^ | | 3x WT, 1x biv.BA.5, 1x XBB | 26 | f | 33 | 333 | 149 | 161 | 263 | 10 | 10 | 52 | 30 | 161 | 263 |
| 26 | XBB/JN.1^2^ | | 4x WT, 1x biv.BA.5, 1x XBB | 61 | f | 33 | n.d. | 3241 | 2471 | 2552 | 528 | 326 | 1398 | 760 | 2471 | 2552 |
| 27 | BA.5/BQ.1^2^ | | 3x WT, 1x biv.BA.5 | 55 | f | 21 | 15 | 11988 | 4204 | 289 | 360 | 142 | 137 | 245 | 4204 | 289 |
| 28 | BA.5/BQ.1^2^ | | 3x WT, 1x biv.BA.1 | 37 | m | 27 | 19 | 1711 | 465 | 93 | 66 | 10 | 63 | 39 | 465 | 93 |
| 29 | BA.5/XBB^2^ | | 3x WT, 1x biv.BA.1 | 59 | m | 161 | 37 | 665 | 346 | 54 | - | - | - | 54 | 346 | 54 |
| 30 | BA.5/XBB^2^ | | 3x WT, 1x biv.BA.5 | 26 | m | 153 | 49 | 1776 | 1526 | 217 | 243 | 65 | 85 | 151 | 1526 | 217 |
| 31 | 1^st^ BA.2, 2^nd^ XBB^3^ | | 3x WT, 1x biv.BA.5, 1x XBB | 63 | m | 34 | 305 | 1608 | 1632 | 1139 | 365 | 299 | 755 | 499 | 1632 | 1139 |
| 32 | 1^st^ BA.5, 2^nd^XBB^3^ | | 4x WT | 54 | f | 414 | 26 | 1264 | 691 | 500 | 207 | 111 | 121 | 226 | 691 | 500 |
| BTI | | Breakthrough infection | | | | |  |  |  |  |  |  |  |  |  |  |
| ^1^dpV | | Days post vaccination | | | | |  |  |  |  |  |  |  |  |  |  |
| ^2^dpI | | Days post infection | | | | |  |  |  |  |  |  |  |  |  |  |
| GMT | | Geometric Mean Titer | | | | |  |  |  |  |  |  |  |  |  |  |
| pVNT | | Pseudovirus neutralization test | | | | |  |  |  |  |  |  |  |  |  |  |
| VNT | | Virus neutralization test | | | | |  |  |  |  |  |  |  |  |  |  |
| WT | | wildtype | | | | |  |  |  |  |  |  |  |  |  |  |
| Biv.BA.5 | | Bivalent BA.5 | | | | |  |  |  |  |  |  |  |  |  |  |
| ^1^ Single-variant infections (BA.2, BA.5, BQ.1.1, XBB) | | | | | | | | | | | | | | | | |
| ^2^ Infections during waves with co-circulating variants where the infecting lineage could not be clearly assigned (XBB/JN.1, BA.5/BQ.1.1, BA.5/XBB) | | | | | | | | | | | | | | | | |
| ^3^ Sequential infections indicating first and second infecting variants (1^st^ BA.2, 2^nd^ XBB; 1^st^ BA.5, 2^nd^ XBB) | | | | | | | | | | | | | | | | |


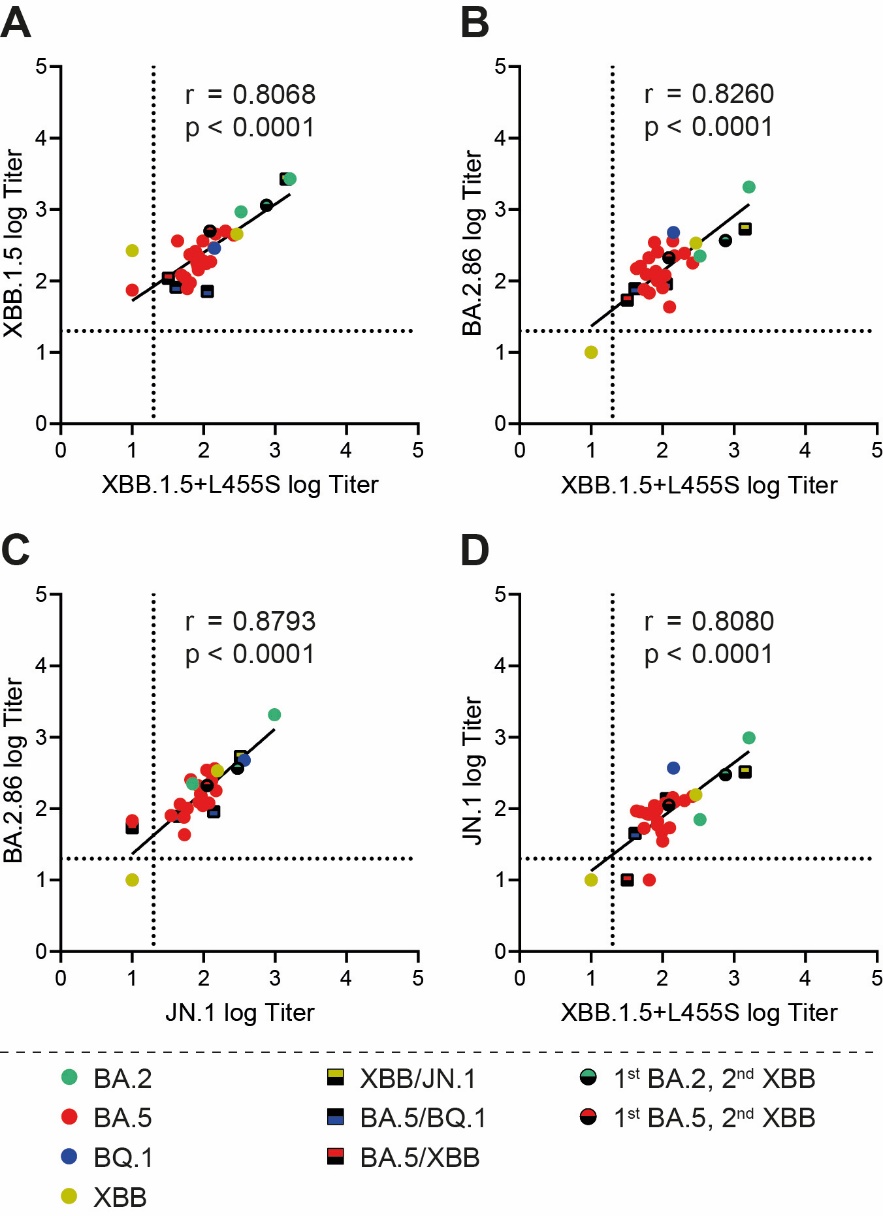


**Figure S2. Pearson´s correlation of geometric mean neutralization titers measured by pVNT against SARS-CoV-2 variants.**Correlation of log10-transformed serum neutralization titers between SARS-CoV-2 XBB.1.5+L455SA and XBB.1.5 (A), XBB.1.5+L455S and BA.2.86 (B), JN.1 and BA.2.86 (C), and XBB.1.5+L455SA and JN.1 (E) as determined by pseudovirus neutralization tests (pVNTs). A total of 31 serum samples were analyzed in both assays. Breakthrough infection variants are indicated by color and symbol. Single BTIs are shown as fully colored circles; co-circulating–variant infections as half-black, half-colored squares; and first and second infections as half-black, half-colored circles (see Table S1 and S2). Dashed lines indicate the cut-off threshold. For negative sera, values were set to half of the cutoff. Data are from at least three independent experiments. Pearson correlation coefficients (r) and p values are indicated.
